# Cell Component and Function of Tumor Microenvironment in Thyroid Cancer

**DOI:** 10.3390/ijms232012578

**Published:** 2022-10-20

**Authors:** Eunah Shin, Ja Seung Koo

**Affiliations:** Department of Pathology, Yonsei University College of Medicine, Seoul 03722, Korea

**Keywords:** cancer associated fibroblast, immune cells, tumor microenvironment, thyroid cancer

## Abstract

Thyroid cancer is the most common cancer in the endocrine system. Most thyroid cancers have good prognosis, but some of them are resistant to treatment or show aggressive behavior. Like other tumors, thyroid cancers harbor tumor microenvironment (TME) composed of cancer associated fibroblasts (CAF) and immune cells. Autoimmune lymphocytic thyroiditis can occur in the thyroid, and it may be associated with cancer development. TME is involved in tumor progression through various mechanisms: (1) CAF is involved in tumor progression through cell proliferation and extracellular matrix (ECM) remodeling; and (2) immune cells are involved in tumor progression through cell proliferation, angiogenesis, epithelial mesenchymal transformation (EMT), and immune suppression. These events are activated by various cytokines, chemokines, and metabolites secreted from cells that comprise TME. This review is focused on how CAF and immune cells, two important cell components of thyroid cancer TME, are involved in tumor progression, and will explore their potential as therapeutic targets.

## 1. Introduction

Thyroid cancer is the most common cancer arising in the endocrine system [1], and its incidence rate is rapidly increasing [2]. Thyroid cancers mostly arise from follicular cells except for medullary carcinoma (MC) that arises from parafollicular c-cells. Thyroid cancers arising from follicular cells are classified as well-differentiated carcinoma, poorly differentiated carcinoma (PDC), and anaplastic (undifferentiated) carcinoma (ATC) according to histology and clinical features. Well-differentiated carcinomas, namely papillary thyroid carcinoma (PTC) and follicular thyroid carcinoma (FTC), comprise 90% of thyroid cancers [3]. Thyroid cancers show various clinical characteristics and treatment effects according to histologic subtypes [4], and PTC and FTC show relatively good prognosis. However, about 10% of PTC and FTC show resistance to radioactive iodine therapy, and ATC and MC, although rare in occurrence, have aggressive features with poor prognosis [5,6]. Treatment modalities for thyroid cancers include surgery, endocrine inhibition therapy, and radio-iodine therapy [7,8]. However, as these therapeutic options also have limitations and complications [9,10], research on new treatment targets is warranted.

Tumor microenvironment (TME) is the non-transformed area around tumor cells, mainly composed of cancer associated fibroblasts (CAF) and immune cells [11,12,13,14]. TME has an impact on tumor progression and treatment response through interactions with tumor cells. Like any other tumors, thyroid cancers have TME as well that affects various aspects of tumor behavior. This review is focused mainly on the cell components of TME in thyroid cancers and how they affect cancer biology. The possible potential of suppressing the crosstalk between TME and tumor cells as a new treatment option is also explored.

## 2. General Characteristics of Tumor Microenvironment

### 2.1. Cancer-Associated Fibroblasts (CAF)

TME is composed of immune cells, fibroblasts, myofibroblasts, endothelial cells, adipocytes, and extracellular matrix (ECM), and CAF are the main cellular component of TME [11,12,13,14]. CAF are larger in cell size, have more cytoplasmic branches, are more potent in proliferation and migration, and can produce more ECM than fibroblasts [11,13,15]. A number of markers, such as α-SMA, FSP-1, FAPα, NG2, PDGFR-β, and prolyl 4-hydroxase, have been explored to identify CAF; however, none of them are specific for CAF [12]. Resident tissue fibroblasts, mesenchymal stem cells, bone-marrow derived fibrocytes, adipocytes, endothelial cells, and epithelial cells are suggested as the origin of CAF, implying that various cells can differentiate into CAF [12,15]. Tumor cells and/or transformed epithelial cells that are destined to be tumor cells produce soluble factors and lipid-based particles, which induce fibroblasts to differentiate into CAF, and then recruit and activate CAF. Activated CAF are involved in tumor cell proliferation, expression of cancer cell stemness, and reprogramming of tumor metabolism through crosstalk such as cell to cell contact between tumor cells and receptor-ligands. CAF are also involved in ECM remodeling, immune modulation, and tumor cell migration and metastasis via EMT [16,17]. Growth factors such as HGF, VEGF, EGF, CTGF, IGF, and NGF, cytokines such as IL-6, IL-11, and IL-17, and chemokines such as CCL7, CCL5, CXCL12, and CXCL7 are produced by CAF in the process of interaction between tumor cells and CAF [18].

### 2.2. Tumor Infiltrating Immune Cells

Immune cells comprising TME can be subgrouped into tumor-associated myeloid cells (TAMC) and tumor-associated lymphoid cells (TALC). TAMC include tumor-associated macrophages (TAM), myeloid-derived suppressor cells (MDSC), tumor-associated neutrophils (TAN), tumor-associated dendritic cells (TADC), and tumor-associated mast cells (TMC). TALC include T cells and NK cells.

#### 2.2.1. Tumor-Associated Myeloid Cells (TAMC)

TAM are the most common and large component among infiltrating immune cells and they are subdivided into M1 (classic) and M2 (alternative) [19,20]. M1 type TAM secrete cytokines such as TNF-α and IL-1, and thus they are associated with Th1 response, intracellular pathogen killing, and antitumor immunity [21,22]. M2 type TAM secrete IL-10 and IL-13, and they are associated with killing and encapsulation of parasites [23], Th2 activation [24], tissue remodeling [25], and tumor induction and growth [26]. Markers for M1 macrophages are CD64, IDO, SOCS1, and CXCL10. Markers for M2 macrophages are MRC1, TGM2, CD23, and CCL22 [27]. It is suggested that TAM are derived from tissue-resident macrophages (TRM) and bone-marrow-derived macrophages (BMDM), and the compositional ratio of TRM and BMDM in TME is different according to the tumor types [28]. The mechanisms by which TAM promote tumor progression are as follows: (1) TAM promote tumor invasion and metastasis by initiating EMT of tumor cells through secretion of signaling molecules such as EGF, SPARC, MMP, CCL2, and CCL18; (2) TAM induce angiogenesis within the tumor by secreting angiogenic factors such as VEGF, PDGF and bFGF; (3) TAM promote and maintain production of cancer stem cells by secreting cytokines such as TNF-α, IL-6, and EGF; and (4) TAM induce immunosuppression by suppressing CD8+ T-cells and NK cells and promoting proliferation of immunosuppressive Treg and MDSC through crosstalk between cytokines, metabolic enzymes and/or surface receptors such as IL-10, TGF-β, CCL2, CCL17, CCL20, CCL22, and PD-L1 [29].

MDSC are heterogeneous and immature myeloid cells, functioning as immune suppressor. The mechanism by which MDSC induce immune suppression is complex. Monocytic-MDSC produce nitric oxide (NO) through gene expression of inducible nitric oxide synthase (iNOS) gene. Granulocytic-MDSC produce ROS and arginase 1, which cause cell cycle arrest by amino acid l-arginine depletion and suppressing T cell receptor (TCR)–chain expression [30]. These NO and ROS induce TCR peroxynitration and T cell apoptosis [31]. MDSC activate Treg by secretion of IL-10, and they also activate Th17 cells by secretion of IL-6 and TGF-β [32]. In addition, when MDSC are activated, COX2 [33] and PD-L1 [34] are expressed, resulting in immunosuppression.

TAN, like TAM, can also differentiate into anti-tumor type (N1) and pro-tumor type (N2) [35,36,37]; N1 TAN have high levels of TNFα, CCL3, and ICAM-1 and low levels of arginase, whereas N2 TAN show high levels of CCL2, CCL3, CCL4, CCL8, CCL12, CCL17, CXCL1, CXCL2, IL-8/CXCL8, and CXCL16 [36,37]. The mechanisms by which N1 TAN show anti-tumor activities are antibody-dependent cytotoxicity by neutrophil elastase (NE), activation of innate and adaptive immune cells such as lymphocytes, NK cells and dendritic cells, and tumor cell suppression through ROS production by increasing NADPH oxidase activity [38,39]. The mechanisms by which N2 TAN induce tumor progression are tumor cell proliferation activated by NE, genetic alteration by ROS, CD8+ T cell suppression by arginase depletion, and immunosuppression by activating immunosuppressive Treg [36].

There are a few subsets of TADC comprising TME. Plasmacytoid dendritic cells (pDC) and pre-dendritic cells (pre-DC) are derived from bone marrow macrophage/dendritic cell (DC) progenitors (MDP), and CD11b + DCs and CD103 + DCs come from pre-DC. Inflammatory DCs (inf-DC) are derived from M-MDSC and monocytes. Therefore, TADC comprising TME are pDC, CD11b + DCs, CD103 + DCs, and Inf-DC [40]. DCs induce immune reaction through antigen presentation, and TADC interfere with the normal development of DCs, their activation and function through following mechanisms: (1) DCs are differentiated to tolerogenic DCs that have low expression of MHC molecule and secrete high level IL-10; (2) IL-10 that is produced by TAM suppresses IL-12 production from CD103 + DC, suppressing T cell activation; (3) accumulation of abnormal lipid within TADC lowers tumor antigen presenting capability of TADC; (4) tumor cell hypoxia, lactic acid accumulation, and low pH suppress TADC response to T cells; (5) IDO produced from pDC promote differentiation of immunosuppressive Treg; and (6) various soluble factors secreted from the tumor cells promote differentiation of inf-DC, which secrete tumor promoting IL-6 and immunosuppressive galectin-1 [40].

TMC play the dual role of tumor progression and tumor suppression. The mechanisms by which TMC play a role in tumor progression are as follows: (1) promotion of angiogenesis by heparin-like molecule, histamine, TNF-α, VEGF, platelet activating factor, IL-8, bFGF and prostaglandin that are secreted from TMC [41,42]; (2) promotion of tumor invasion and metastasis through extracellular matrix degradation by chymase, cathepsin G, carboxypeptidase, gelatinase A and B that are secreted from TMC [43,44]; and (3) immune suppression by secreting inhibitory cytokine IL-10 and maintaining Treg activation that is important in immune tolerance [45]. On the other hand, the mechanisms by which TMC play a role in tumor suppression are exocytosis of granules that contain serine protease and tumor cell cytotoxicity through receptor-ligand binding through TNF-α and FasL [46].

#### 2.2.2. Tumor-Associated Lymphoid Cells (TALC)

T-cells and NK cells are two main types of TALC, and T-cells are classified into Treg, cytotoxic T cells, and helper T cells according to cell function. Treg can be found in TME, and they suppress anti-tumor immune reaction by recognizing TCR-antigen complex on MHC molecule [47]. The chemokine-chemokine receptors that are involved in recruiting Treg around tumor area are CCR4-CCL17/22, CCR10-CCL28, and CXCR4-CXCL12 [48]. Cytotoxic T cells are activated by recognizing tumor antigen presented by MHC class I molecule, bind to tumor cells and secrete granzyme B and perforin, and eventually induce tumor cell apoptosis [49]. Helper T cells are subdivided into Th1 and Th2. Th1 activate anti-tumor immune reaction by secreting IL-2 and IFN-γ whereas Th2 suppress anti-tumor immune reaction by secreting IL-4, suppressing NK cells, and lowering tumor antigen expression [50]. NK cells can be further classified into two subtypes, CD56^dim^CD16^+^ NK cells and CD56^bright^CD10^−/low^ NK cells. CD56^dim^CD16^+^ NK cells clear tumor cells through cytotoxic activity, and CD56^bright^CD10^−/low^ NK cells can suppress immune reaction by secreting IL-13 [51,52]. NK cells are activated by Fas ligand-Fas, secrete granzyme B and perforin, and induce tumor cell apoptosis, eventually inducing tumor cell necrosis by ADCC through antibody-FcR complex [53]. The mechanisms by which tumor cells interfere with NK cell function are as follows: (1) secretion of immunosuppressive factors such as TGF-β, IOD, and arginase-1; (2) decreased expression of MHC class I molecules on tumor cells; and (3) inhibition of ligand upregulation and activation of Treg [54].

One of the important mechanisms by which TALC promote tumor progression is immune check point modulation. Among immune check points, one that is important in tumor cells is inhibitory immune check points, namely CTLA-4 and PD-1. CTLA-4 is expressed on the surface of activated lymphocytes, binding to CD80 and CD86 on the surface of APC. This binding suppresses binding of CD80 and CD86 with CD28, suppressing T cell activation [55]. PD-1 is expressed on the surface of activated T cells, B cells, and NK cells, binding with PD-L1 and/or PD-L2 and suppressing CTL function [56].

## 3. Cell Component and Function of Tumor Microenvironment in Thyroid Cancer

**Figure 1:**
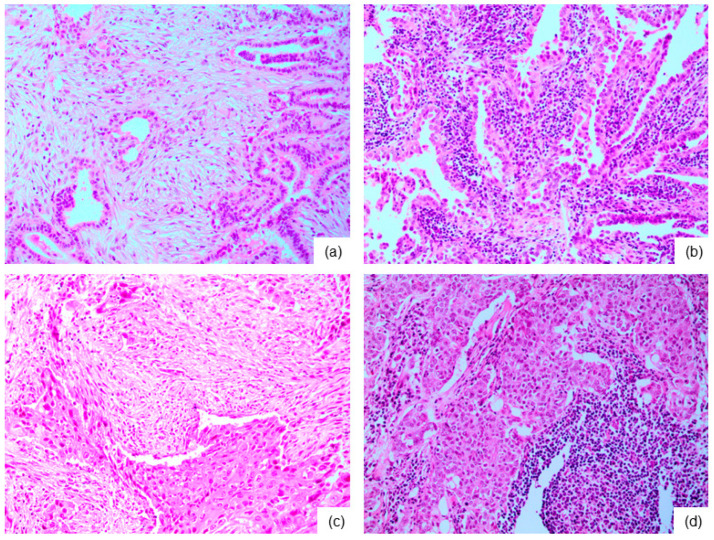
Tumor stroma in thyroid cancer. Papillary thyroid carcinoma (PTC) shows desmoplastic tumor stroma mainly composed of caner-associated fibroblasts (CAF) (×100) (**a**). Prominent immune cells are noted in PTC subtype such as Warthin-like variant (×100) (**b**). Fibrous stroma composed of CAF is noted in anaplastic thyroid carcinoma (×100) (**c**). Immune cells stroma is present in poorly differentiated thyroid carcinoma (×100) (**d**).

TME in thyroid cancer, like TME in other tumors, are composed of CAF and immune cells and affect tumor biology of tumorigenesis, growth, and progression (Figure 1). First, thyroid cancer can have abundant fibrous stroma, especially in PTC [57,58,59] and ATC [60], suggesting the possible presence of abundant CAF. As the thyroid can often harbor chronic lymphocytic thyroiditis (CLT), many studies have reported that thyroid cancer, especially PTC, is related with CLT [61,62]. Warthin-like variant and diffuse sclerosing variant are histologic subtypes of PTC that are accompanied by an abundance of lymphoplasma cells [63,64]. PTC with CLT has good prognosis with limited disease progression [65,66]; however, PTC with tumor infiltrating lymphocytes is reported to be found at higher cancer stage and have more frequent lymph node metastases [67]. Lymphocyte density is correlated with lower tumor recurrence and longer OS in PTC [65]. In silico analysis study including 799 PTC and 194 normal thyroid samples has found higher levels and proportions of M2 macrophages, Tregs, monocytes, neutrophils, DCs, mast cells (MCs), and M0 macrophages in PTC than in normal thyroid tissues, and also in advanced PTC than in early PTC. It has also found more advanced stages, larger tumor sizes, greater lymph node metastases, higher tall-cell PTCs, lower follicular PTC proportions, more BRAF mutations, and fewer RAS mutations in high-immunity group [68], suggesting that thyroid cancer harbors an abundance of immune cells in TME that affects tumor biology.

### 3.1. CAF in Thyroid Cancer

**Figure 2:**
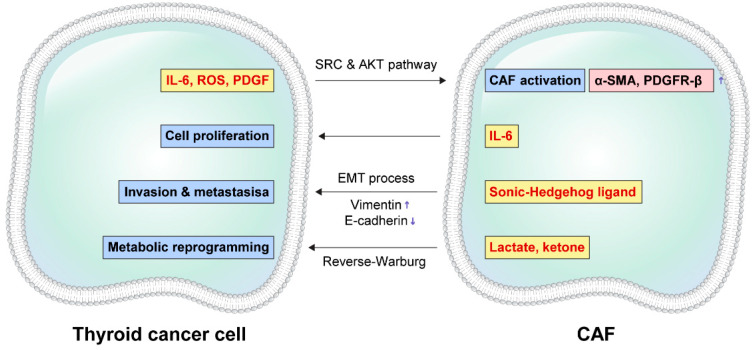
Interaction between cancer cells and caner-associated fibroblasts (CAF) in thyroid cancer. The expressions of CAF markers, α-SMA and PDGFR-β, are increased by SRC and AKT pathway activated by IL-6, ROS, PDGF that are secreted by thyroid cancer cells. SRC and AKT pathway differentiates and activates CAF. IL-6 secreted by CAF promotes tumor cell proliferation, and Sonic-Hedgehog ligand secreted by CAF promotes cancer cell invasion and metastasis through EMT process. In this EMT process, the expression of vimentin is increased and the expression of E-cadherin is attenuated. Moreover, lactate and ketone produced by glycolysis in CAF are transported to tumor cells and used in energy production by TCA cycle. This metabolic reprogramming is called the reverse-Warburg effect.

A few studies on thyroid cancer tissues have found expression of CAF-related proteins in thyroid cancer stroma [69,70,71], and have revealed that certain CAF-related protein expression is related with cervical lymph node metastasis [69], histologic subtype, BRAF mutation, and prognosis [70]. Increased fibroblast infiltration around the tumor area has been found not only in human thyroid cancer tissues but also in mouse thyroid cancer models [72,73], both induced by oncogenic BRAF. Soluble factors produced by cancer cells are important in CAF generation and/or recruitment in thyroid cancer. It has been found in in vitro co-culture study that conditioned media by human ATC cells (8505c and KTC-2) are involved in reprogramming of thyroid fibroblast phenotype to CAF phenotype [74], and conditioned media by thyroid cancer cells that are induced by BRAF mutation promote CAF proliferation and migration in thyroid cancer mouse model [73]. Conditioned media by human ATC cells (8505c and KTC-2) activate Src and Akt pathway and accelerate human fibroblast proliferation, with increase in the expression of CAF-related markers, namely α-SMA and PDGFR-β [74]. Molecular candidates responsible for the reprogramming of fibroblast phenotype to CAF phenotype are IL-6, ROS, and PDGF [74]. The molecular mechanisms through which CAF affect tumor biology of thyroid cancer are as follows (Figure 2): (1) CAF promote thyroid cancer cell proliferation. Thyroid follicular cell (FRTL-Tc) injection in a mouse model did not develop thyroid cancer, but follicular cell (FRTL-Tc) injection with fibroblast caused tumor development [75]. Moreover, conditioned media by CAF in in vitro co-culture study promoted thyroid cancer cell proliferation, showing a high level of mitogen secretion, IL-6, in CAF [74]; (2) CAF promote migration and invasion in thyroid cancer cells through EMT. In in vitro co-culture study, conditioned media by CAF increased the expression of vimentin, an EMT marker, in thyroid cancer cells, and decreased the expression of E-cadherin, an anti-EMT marker, resulting in increased invasion activity [74]. Moreover, Sonic-Hedgehog ligand secreted by CAF induces ATC cell migration [76]; and (3) CAF induce metabolic reprogramming in thyroid cancer cells. One theory describing metabolic characteristics of cancer cells, the so-called Warburg effect, explains that cancer cells produce energy through glycolysis rather than mitochondrial oxidative phosphorylation [77]. However, the reverse-Warburg effect model describing metabolic crosstalk between cancer cells and TME explains that glycolysis in CAF produces lactate, ketone body, and pyruvate that are transported into cancer cells by lactate shuttle, resulting in energy production through mitochondrial oxidative phosphorylation [78,79,80]. An in vitro cell line study has found that CAF exposed to conditioned media by human ATC cells (8505c and KTC-2) have high levels of glycolysis related molecules, GLUT-1 and LDH-A, by HIF-1α modulation, and glucose uptake and glycolysis are increased as a result [74]. Additionally, an IHC study has confirmed that the expression of MCT4, a lactate shuttle, is increased in the stroma of ATC tissue [81]. CAF promote tumor progression in thyroid cancer through these various mechanisms, and they are related with dedifferentiation and aggressiveness. When mRNA-based CAF gene signature was investigated using thyroid cancer public data, high CAF score was correlated with anaplastic phenotype, poor prognosis, high genetic mutation, and oncogenic signaling pathway [82].

### 3.2. Immune Cells in Thyroid Cancer

**Figure 3:**
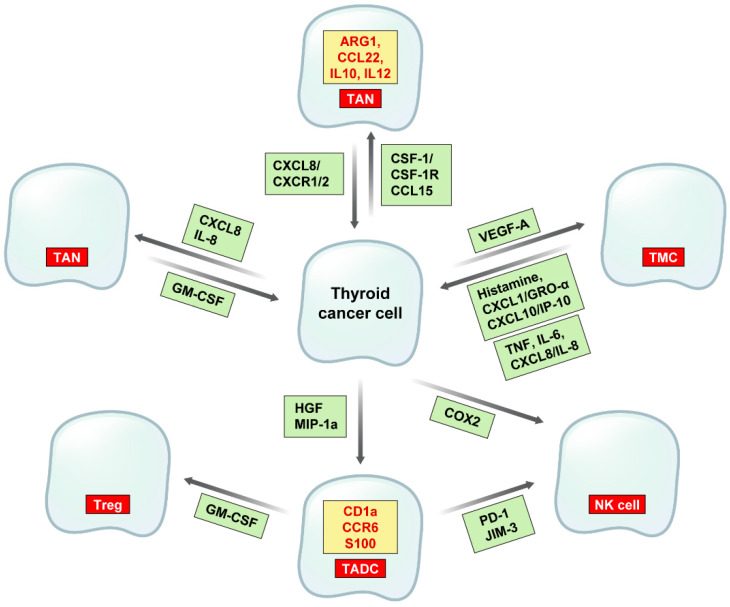
Interaction between cancer cells and immune cells in thyroid cancer. CSF-1 and CCL15 produced by thyroid cancer cells recruit TAM, which produce CXCL8 that binds to CXCR1/2 and promotes tumor progression. TAM in thyroid cancers are M2 types that express ARG1, CCL22, IL10, and IL12. CXCL8 and IL-8 recruit TAN, which produce GM-CSF that is involved in tumor progression. VEGF-A recruits TMC, which produce histamine, CXCL1/GRO-α, and CXCL10/IP-10 that promote cancer cell proliferation. TMC also produce TNF, IL-6, and CXCL8/IL-8 that are involved in EMT. HGF and MIP-1a recruit TADC, which recruit Treg by ICOS, and PD-1 and JIM-3 recruit NK cells. TADC in thyroid cancers express CD1a, CCR6 and S100, and thyroid cancer cells produce COX-2 to recruit NK cells.

Immune cells in TME in thyroid cancer affect cancer cell biology by various crosstalks (Figure 3). IHC has revealed the presence of CD68 positive TAM in thyroid cancer, and macrophage infiltration rate was significantly increased in PTC compared to in benign tumors and was correlated with lymph node metastasis and poor prognosis [83,84,85,86,87]. TAM density differs according to histologic subtypes; ATC has the highest TAM density and poor prognosis [84]. TAM density is correlated with invasiveness and metastasis in PTC, as a result of CXCL8 secreted from TAM binding with CXCR1/2 secreted from PTC [87]. As in PTC, TAM is related with capsular invasion and extrathyroidal extension in PDTC [85]. PTC employs CSF-1/CSF-1R signaling to recruit TAM as the tumor progresses [72]. TAM infiltration rate is higher in FTC than in follicular adenoma, and CCL15 is involved in this process [88]. TAM in BRAF V600E-related PTC are M2 phenotypes that show high levels of ARG1, CCL22, and IL-10 and a low level of IL-12 [87]. However, controversy exists regarding the association of TAM with prognosis, because there are reports that assert that the number of tumor-infiltrating macrophages is correlated with longer DFS in thyroid cancer patients [89]. The preoperative level of circulating MDSC was higher in thyroid cancer patients than in those with benign thyroid disease [90,91], and the circulating MDSC level was correlated with the aggressiveness of differentiated thyroid cancers [91]. There was no significant correlation between MDSC density and clinicopathologic factors in studies on MDSC in thyroid cancer tissues [89]. Studies on neutrophils in thyroid cancers mainly involved peripheral blood neutrophil-to-lymphocyte ratio (NLR). NLR was correlated with treatment response in thyroid cancers in that NLR was significantly decreased when treatment response was good and thus good prognosis, but NLR was significantly increased when treatment response was not good [92]. Increased NLR was correlated with poor treatment response in multivariate analysis, suggesting that the increased systemic inflammation after treatment incurred poor treatment response [92]. There are, however, reports that NLR is not correlated with benign or malignant tumor behavior and nor with disease prognosis [93]. TAN recruited by CXCL8/IL-8 secrete GM-CSF and promote tumor cell survival and tumor progression, evidenced by research involving human thyroid cancer tissues that have shown correlation between TAN density and tumor size [94]. PTC has increased TADC [95,96], especially CD1a positive TADC, that correlate with improved DFS [97]. TADC recruitment in PTC is due to MIP-1a secreted by HGF, and CCR6 expressing TADC clear damaged thyroid cells [98]. S100 positive TADC in PTC are not related with DFS [95], thus there are differences between TADC phenotypes. TADC are also involved in immune escape in PTC by transforming CD4 positive T cells to FOXP3 positive Treg [96].

Mast cells are one of the immune cells that have been researched more frequently in PTC than other immune cells. IHC for tryptase, a mast cell marker, in human thyroid cancer tissues has revealed that 95% of PTC show mast cells and the amount of TMC was correlated with extrathyroidal tumor extension [99]. TMC are also found in PDTC and ATC, in which TMC density was correlated with tumor invasiveness [100], and TMC density was significantly increased in FVPTC than in follicular adenoma [101]. Thyroid cancer cell line study has shown that VEGF-A is secreted from thyroid cancer cells and it promotes mast cell chemotaxis. Thus, it is suggested that VEGF pathway is involved in TMC recruitment in PTC [99]. TMC can be found in various thyroid cancer tissues and they are related with tumor aggressiveness, due to various soluble factors they produce, which are mostly non-IgE related. Histamine, IL-6, IL-1, TNF-α, and chemokines such as CXCL1/GRO-α, CXCL8/IL-8, and CXCL10/IP-10 are secreted from mast cells when thyroid cancer cells are activated [89]. First, TMC promote thyroid cancer cell proliferation by producing histamine that binds to H1 and H2 receptors in PTC tumor cells. A mouse xenograft study has suggested histamine, CXCL1/GRO-α and CXCL10/IP-10 as TMC secreting factors that are important in thyroid cancer cell proliferation [29]. Second, TMC initiate EMT, evidenced by studies using thyroid cancer cell lines (PTC, FTC, and ATC) that showed an EMT process of morphological change of thyroid cancer cells to spindle cells, increased EMT markers, and decreased epithelial markers when the cancer cells were exposed to conditioned media by activated mast cells [100]. TMC secreting factors that are essential in this EMT process are TNF, IL-6 and CXCL8/IL-8. CXCL8/IL-8 is especially important in the process [100]. Third, TMC induce cancer cell stemness, showing enhanced stemness features when thyroid cancer cells are exposed to conditioned media by mast cells or when exposed to recombinant CXCL8/IL-8 [100]. Such EMT and cancer cell stemness by TMC in thyroid cancer cells are activated by AKT/SLUG pathway. In IHC study of tryptase, a mast cell marker, and OCT-4, a stem cell marker, using human PTC tissues, there was a significant positive correlation between TMC density and OCT-4 expression. TMC density and OCT-4 expression were also correlated with higher T stage [100].

Treg, a subtype of T-cell, are found in PTC tissues in a significantly larger number than in benign goiter [96,102], and Treg in PTC are correlated with extrathyroidal extension and LN metastasis [102]. Additionally, Treg are found in abundance in metastatic lymph nodes of PTC, and they are correlated with recurrent PTC [103]. Treg are also related with the aggressiveness of papillary microcarcinoma, due to the immune suppression incurred by activation of Treg differentiation promoted by increased expression of IDO1 in tumor cells [104]. The mechanism by which Treg are recruited in PTC is transformation of CD4+ T cells into FoxP3+ICOS+Treg through inducible costimulatory (ICOS) ligand by plasmacytoid DC in PTC microenvironment [96]. In IHC study of FoxP3 involving thyroid cancer tissues (PTC and FTC) and benign thyroid tissue, FoxP3 expression was correlated with tumor aggressiveness, and tumor size was inversely correlated with FoxP3 expression [105]. New subtypes of T cells are suggested in thyroid cancer; (1) DN T cells (CD3+CD4-CD8-double negative T cells) comprise the main population of T cells in PTC TME, suppressing proliferation of activated T cells and hence decreased cytokine production [106]. CD4+IL17+ T cells (Th17) are more abundant in DTC than in benign thyroid tissue [89], and Th17 density is significantly higher in thyroid tumor than in normal thyroid tissue. Th17 density is also correlated with the serum level of IL-17 [107]. In contrast with Treg, CD8+ T cell concentration is lower in PTC patients, and CD8+ T cell/FoxP3+ Treg ratio is inversely correlated with tumor size [67]. It has been reported that CD8+ T cell infiltration in differentiated thyroid cancer is correlated with increased tumor recurrence [108], but there are reports of the contrary, that CD8+ T cell infiltration is correlated with improved DFS [89], because CD8+ T cell is in anergy without granzyme B [108]. In a gene informatics study, CLDN10 is related with CD8+ T cell infiltration in PTC and associated with good prognosis [109].

In a flow cytometry study, NK cells are significantly more abundant in PTC than in benign nodular goiter, but more abundant in early PTC than in advanced PTC [110]. NK cell dysfunction through PD-1/TIM-3 pathway in TME immune cells [111], and decreased expression of activated receptors for NK cells, such as NGK2D, by increased COX2 expression in tumor cells are two mechanisms by which tumor cells avoid cell lysis by NK cells in ATC [112]. ATC cells are sensitive to tumor cell lysis by ULBP2/5/6 and CXCR3-positive NK cells [112], and injection of NK cells in a mouse model of ATC pulmonary metastasis suppressed the growth of pulmonary metastatic tumor [113].

### 3.3. Immunologic Classification of Thyroid Cancer

Various immune cell infiltration in thyroid cancers is affected by various factors, but especially so by tumor genomic features. Thyroid cancers are classified according to histologic classification, and PTC can be further classified as BRAFV600E-like (BVL)-PTC and RAS-like (RS) PTC according to molecular classification on the basis of TCGA data [114]. BVL PTC and RS PTC have different genetic, epigenetic, and proteomic features, and development of BRAFV600E-RAS score (BRS) system can help elucidate molecular characteristics of PTC [115]. Certain genetic mutations in thyroid cancers can have various effects on immune cells. BRAF V600E mutation in PTC is associated with increased DC, TAM and mast cells, and also with elevated expression levels of CTLA-4, PD-L1 and PD-L2 [116]. It is also associated with Treg and immunosuppressive TAM [117]. TERT promotor methylation and BRAF V600E mutation are associated with PD-L1 expression in primary thyroid cancer [118]. RET/PTC3 genetic mutation promotes IDO1 expression through STAT1-IRF1 pathway [119]. IDO1 suppresses activated T cell proliferation and promotes Treg differentiation, controlling immune cell population phenotype in RET mutated thyroid cancer. Accordingly, such immunologic features can serve as classification criteria for thyroid cancers. A large scale immunogenomic analysis using TCGA data classified tumors into six immune subtypes (C1-C6) of wound healing, IFN-γ dominant, inflammatory, lymphocyte depleted, immunologically quiet, and TGF-β dominant [120]. These subtypes differ in lymphocyte/macrophage signature, Th1:Th2 ratio, intratumoral heterogeneity status, aneuploidy, neoantigen level, cell proliferation level, immunomodulatory gene expression, and prognosis. PTC is classified as inflammatory subtype, with balanced macrophage/lymphocyte ratio, low intratumoral heterogeneity, low aneuploidy level, low level of somatic copy number alteration, low cell proliferation, and high expression of genes representing Th17 differentiation. Another study using TCGA data and ESTIMATE datasets found 793 differentially expressed genes in thyroid cancer TME, and these genes were associated with immune score and stromal score [121]. Immune score and stromal score can be obtained from ESTIMATE website and they can predict immune cell and stromal cell infiltration in TME. Immunoscore has been developed in PTC, which can be obtained from the amount of CD3+ and CD8+ T cell population from the center of the tumor (CT) and from the invasive margin (IM). Immunoscore ranges from immunoscore 0 (I0) to immunoscore 4 (I4). [122]. I0 means low CD3+ and CD8+ T cell density in CT and IM, I4 means high CD3+ and CD8+ T cell density in CT and IM. In a study using TCGA data of PTC, immunoscore was negatively correlated with thyroid differentiation score (TDS), and high immunoscore was found in BRAFV600E mutated PTC [116]. An NGS study involving thyroid cancer tissues (25 PTC, 14 PDTC, and 13 ATC) and normal thyroid tissues (7 NT) has reported two clusters with immune-related genes [123]. The first cluster is composed of ATC and some of PTC showing high regulation of immune-related genes, and the second cluster is composed of PDTC and a part of PTC group showing low regulation of immune-related genes. According to the study results, the TAM and CD8+ T cell density in TME is highest in ATC, followed by PTC and PDTC. Another study has suggested four categories of thyroid cancer (hot, altered-immunosuppressed, altered-excluded, and cold) based on immune cell density and immunoscores [124]. Hot tumor is characterized by high T cell and CTL infiltration in CT and IM, high immunoscore, and suppressed T cell function due to activated immune checkpoints such as PD-1, CTLA-4, TIM3, and LAG3. Altered-immunosuppressed tumor shows an intermediate degree of T cell and CTL infiltration and immunoscore, T cell checkpoints (PD-1, CTLA-4, TIM3, and LAG3), immune suppressive cells (MDSC and Treg), and inhibitory cytokines (TGF-, IL-10 and VEGF). Altered-excluded tumor does not harbor T cell infiltration in CT, shows intermediate immunoscore in IM, and is associated with oncogenic activation, aberrant vasculature/stroma, and hypoxia. Cold tumor has the lowest immunoscore, no T cell infiltration in CT and IM, low tumor mutation burden, low antigen presentation, and T cell insensitivity. Another study has subgrouped thyroid cancers into ATC-like and PDTC-like phenotypes on the basis of NGS and immunoscore data with CD3+ cell density [125]. ATC-like tumor shows high levels of T cell infiltration, chemo-cytokines (CCL2, CCL3, CCL4, CCL5, CXCL9, and CXCL10), and immune checkpoints. PDTC-like tumor is characterized by low levels of T cell infiltration, chemo-cytokines and immune checkpoints. In this study, 50% of PTC were ATC-like and the rest were PDTC-like [125].

## 4. Future Targets for Thyroid Cancer Treatment

As has been described heretofore, many preclinical and clinical studies have been carried out on CAF and immune cells to explore their potential as treatment targets. CAF and immune cells are important components of TME in thyroid cancers and they play important roles in tumor biology.

### 4.1. Targeting CAF for Thyroid Cancer Treatment

CAF can be a good treatment target because they are genetically stable, they are important in maintaining ECM framework of cancer, and they are major barriers to cancer cells against anticancer drug. There are a few general strategies in using CAF as a cancer treatment target; (1) targeting biophysical stromal barrier in order to effectively transport drugs, (2) suppressing molecules secreted from CAF that activate cancer cells, (3) blocking ECM component in order to lower adhesion-induced signaling, and (4) suppressing CAF in order to suppress CAF downstream pathway. There are four strategic categories of CAF inhibitors (Table 1): (1) When the stromal barrier is targeted, the targets are TGF-β, MMPs, and Hedgehog which suppressed the tumorigenesis, migration, and invasion in ATC cells (8505C and/or SW1736) when TGF-β1 was inhibited [126,127]. BB94, an MMP inhibitor, suppressed migration and invasion of ATC cells [127], and when minocycline, an MMP inhibitor, was injected with manumycin and paclitaxel in ATC xenograft study, the tumor size was smaller than when single drug was administered [128]. When cyclopamine, a Hedgehog inhibitor, was administered, the expression of CSC-related transcription factors, B lymphoma Mo-MLV insertion region 1 homolog (BMI1) and SRY-Box Transcription Factor 2 (SOX2), were decreased and the growth of CSC-derived tumor xenografts was inhibited [129]. (2) When CAF secreted molecules are suppressed, the potential targets are CTGF, HGF-c-MET pathway, and CXCR4-CXCL12 axis. When PHA665752, a c-met inhibitor, was administered in PTC cells, HGF dependent cell growth, cell survival, cell invasion, and migration were suppressed by inhibition of c-MET phosphorylation [130]. c-MET inhibitors (tivantinib and crizotinib) suppressed cell growth in 50% of thyroid cancer cell lines [131]. AMD3100, a CXCR4 antagonist, inhibited tumor cell proliferation, invasion and xenograft tumor formation in PTC tumorigenic cell line (BHP10-3M) [132], and BAY11-7082, a CXCR4-CXCL12 axis inhibitor, suppressed CXCL12-CXCR4-induced migration, invasion, and EMT processes by inhibiting NF-κB signaling pathway in PTC cell line (B-CPAP) [133]. (3) When interaction with ECM is suppressed, the key targets are β-integrin and CD44. T315, an integrin-linked kinase inhibitor in thyroid cancer, inhibits thyroid cancer cell migration and shows cytotoxicity even in a very low level [134], and QLT0267, an integrin-linked kinase inhibitor, suppresses tumor cell growth and decreases xenograft tumor size in thyroid cancer cells, especially in ATC [135]. (4) When CAF itself and differentiation into CAF are suppressed, the key targets are FAPα, PDGFR kinase, and VDR ligand, but there has been no report on thyroid cancer.

### 4.2. Targeting Immune Cells for Thyroid Cancer Treatment

There are three strategies in targeting immune cells for cancer treatment in general: (1) tumor vaccines using specific tumor-associated antigen (TAA); (2) adoptive cell therapy using immunocompetent cells segregated from the tumor; and (3) immune checkpoint suppression that increases tumor suppressive ability of immune cells. Studies on immune therapy against thyroid cancer have mainly involved advanced cancer that do not respond to classic treatment or show treatment resistance (Table 2). NY-ESO-1 is a TAA discovered in thyroid cancer, especially in MTC [137,138]. MTC secretes carcinoembryonic antigen (CEA), and Yeast-CEA (GI-6207) vaccine was studied in a clinical trial on metastatic MTC [139]. Neo-antigen that can be used for tumor vaccine is mostly found in abundance in ATC that usually has a high level of mutation burden, and thus tumor vaccine may be more effective in ATC [115]. Oncolytic virus (OV) vaccine that initiates systemic antitumor immunity by tumor cell lysis has been tried in thyroid cancer, and dl922-947, an OV, not only suppressed tumor growth in ATC mouse model but also switched M2 TAM to M1 TAM [140,141,142]. Adoptive cell therapy for thyroid cancer involved injection of tumor-lysate-pulsed DC in advanced PTC, FTC, and MTC, and resulted in palliation of symptoms [143,144]. In a preclinical study of chimeric antigen receptor (CAR)-T cell therapy targeting ICAM-1 in PTC and ATC, tumor growth was suppressed effectively and patient survival improved [145,146]. Monoclonal antibodies for CTLA-4, PD-L1, and PD-1 are key immune checkpoint inhibitors, and when pembrolizumab, a PD-1 inhibitor, was administered in advanced PTC and FTC with PD-L1 expression, there was no major side effect and a few patients experienced symptom relief [147]. In addition, when pembrolizumab was administered with tyrosine kinase inhibitor in ATC patients showing PD-L1 expression, patient survival was extended [148]. The tumor size was decreased and survival period was extended in murine ATC model when anti-PD-1/PD-L1 antibody was administered with BRAF inhibitor [149]. PD-1 blockade together with CAR-T therapy for ICAM-1 in ATC showed tumor suppression and better survival in a mouse xenograft model than CAR-T therapy alone [150].

## 5. Conclusions

Like in other tumors, TME is important in tumorigenesis and tumor progression in thyroid cancer. CAF, one of the main cellular components of TME, are more abundant in fibrous tumor stroma of PTC and ATC among thyroid cancers. Types and functions of CAF have been studied in breast and pancreatic cancers, but not much in thyroid cancers. However, studies to date have reported that CAF can promote thyroid cancer proliferation and progression, and thus CAF suppression may be targeted for treatment in thyroid cancers, especially for refractory thyroid cancers and ATC that does not have effective treatment modality. Studies on CAF inhibitors in thyroid cancers are rather primitive yet and most are preclinical studies, so further studies are warranted. Effective suppression of CAF calls for understanding of specific CAF markers, but as aforementioned, CAF have various cell origins and harbor various markers that have plasticity in phenotypes. This CAF phenotype plasticity is a major barrier in applying CAF inhibitors in thyroid cancers, and thus further research is necessary.

Immune cells, another main cellular component in TME along with CAF, are also important in tumor biology and hence they may be possible treatment targets. A few points should be considered when immune cells are used for thyroid cancer treatment: (1) There are various types and differentiation status. Some studies have reported immune cells to be tumor progressor and some to be tumor suppressor. The role of immune cells in thyroid cancers according to immune cell type and differentiation status has to be studied further and single cell analysis would be important to meet this end. (2) Immune status of thyroid cancers should be considered. As aforementioned, when thyroid cancers are subclassified according to the genetic and immune features, types whose immune system is activated (ATC-like, hot, and altered-immunosuppressed type) and those whose immune system is not activated (PDTC-like, cold, and altered-excluded type), the former recover antitumor immune response by clearing away immunosuppressive signals or immune suppressing cells. The latter, on the other hand, are directed at tumor killing by immune cells through immune cell recruitment to around the tumor cells. (3) Biomarkers that are most effective for immunotherapy should be discovered. Possible biomarkers for cancer immunotherapy in general are PD-L1, tumor mutation burden (TMB), microsatellite instability (MSI), specific gene mutation, immunogenic neo-antigen, and gut microbiome [151], and possible application of these biomarkers for immunotherapy in thyroid cancers should be explored in further studies. PD-L1, for example, is an FDA-approved biomarker for immunotherapy, but its clonal types, interpretation method, and positive criteria are all differently established according to cancer types. Therefore, it is essential to discover effective biomarkers for targeted immune therapy that can be tailored for thyroid cancers.

## Figures and Tables

**Table 1:** CAF inhibitors in thyroid cancer.

Drug	Target	Cancer Type	Study Group	Effect	Reference
Stromal barrier target agents					
TGFβ inhibitor	TGFβ	ATC	Cell line	tumorigenesis↓ migration and invasion ↓	[136] [126,127]
BB94	MMP	ATC	Cell line	migration and invasion ↓	[127]
monocycline	MMP	ATC	Mouse xenograft	xenograft tumor size ↓	[128]
cyclopamine	Hedgehog	ATC	Cell line, mouse xenograft	xenograft tumor size ↓	[129]
CAF secreting factor inhibitors					
PHA665752	c-met	PTC	Cell line	cell growth, cell survival, cell invasion, and migration ↓	[130]
tivantinib and crizotinib	c-met		Cell line	cell proliferation ↓	[131]
AMD3100	CXCR4	PTC	Cell line (BHP10-3M), mouse xenograft	cell proliferation, invasion and xenograft tumor formation ↓	[132]
BAY11-7082	CXCR4-CXCL12 axis	PTC	Cell line (B-CPAP)	migration, invasion, and EMT processes ↓	[133]
CAF-ECM cross-talk inhibitors					
T315	integrin-linked kinase		Cell line	cell migration ↓, cell death ↑	[134]
QLT0267	integrin-linked kinase	ATC	Cell line, mouse xenograft	cell proliferation, and xenograft tumor formation ↓	[135]

**Table 2:** Immune cell modulator in thyroid cancer.

Drug	Target	Cancer Type	Study Group	Effect	Reference
Cancer vaccine					
GI-6207	Yeast-CEA	MTC	Metastatic MCT	Ongoing clinical trial	[139]
dl922-947	oncolytic virus (OV)	ATC	Mouse model	Cell growth ↓, M2 TAM → M1 TAM	[140,141,142]
Adoptive cell therapy					
tumor lysate-pulsed DC		PTC, FTC, and MTC	Advanced PTC, FTC, and MTC	Symptom relieved	[143,144]
CAR-T cell	ICAM-1	PTC and ATC			[145,146]
Immune checkpoint inhibitors					
pembrolizumab	PD-1	PTC and FTC	advanced PTC and FTC	Patient survival time ↑	[147,148]
anti-PD-1/PD-L1 antibody	PD-1/PD-L1	ATC	Mouse model	Tumor size ↓, survival time ↑	[149,150]
